# Impact of Teleconsultation before Endoscopic Submucosal Dissection on Quality of Care and Environmental Sustainability: A French Multicenter Study

**DOI:** 10.1055/a-2932-0269

**Published:** 2026-08-19

**Authors:** Elena De Cristofaro, Amina Abdulle, Vincent Lepilliez, Bertrand Brieau, Thibault Degand, Jérôme Rivory, Alexandru Lupu, Florian Rostain, Frederic Moll, Jean-Baptiste Chevaux, Romain Legros, Nicolas Musquer, Hugo Lepetit, Sarah Leblanc, Marion Schaefer, Yann Le Baleur, Raphaelle Grau, Geoffroy Vanbiervliet, Edouard Chabrun, Arthur Berger, Philippe Leclercq, Ludovic Caillo, Antoine Debourdeau, Paul Doumbe-Mandengue, Gabriel Rahmi, Lucille Quénéhervé, Lucile Héroin, Xavier Dray, Thimothée Wallenhorst, Joel Lacroute, Ariane Vienne, Jérémie Jacques, Mathieu Pioche

**Affiliations:** 1 Gastroenterology UnitPoliclinico Universitario “Tor Vergata”RomeLazioItaly; 2 Gastroenterology Unit 9314 University of TurinTurinPiedmontItaly; 3 Department of Gastroenterology and Endoscopy 89686 Hôpital Privé Jean MermozLyonAuvergne-Rhône-AlpesFrance; 4 Department of Gastroenterology and Endoscopy 84352 Clinique Jules VerneNantesPays de la LoireFrance; 5 Department of Gastroenterology and EndoscopyDijon University HospitalDijonFrance; 6 Department of Gastroenterology and EndoscopyHôpital Edouard Herriot, Hospices Civils de LyonLyonFrance; 7 Service HépatoGastroEntérologie, Brabois Adultes 26920 CHRU de NancyNancyGrand EstFrance; 8 Department of Gastroenterology and EndoscopyDupuytren University HospitalLimogesAquitaine-Limousin-Poitou-CharentesFrance; 9 Department of Gastroenterology 26922 Centre Hospitalier Universitaire de NantesNantesPays de la LoireFrance; 10 Department of Gastroenterology and Endoscopy 89686 Hopital Prive Jean MermozLyonFrance; 11 Department of Gastroenterology and EndoscopyHospital Saint JosephParisParisFrance; 12 Department of Gastroenterology and Endoscopy 36609 Hôpital Edouard HerriotLyonAuvergne-Rhône-AlpesFrance; 13 Department of Gastroenterology and EndoscopyNice University HospitalNiceFrance; 14 Department of Gastroenterology and EndoscopyClinique de l’AnjouAngersAquitaineFrance; 15 Department of Gastroenterology and Endoscopy 36836 Centre Hospitalier Universitaire de BordeauxBordeauxAquitaineFrance; 16 Department of Gastroenterology 74984 Groupe Sante CHCLiègeBelgium; 17 Department of Gastroenterology and Endoscopy 36672 Centre Hospitalier Universitaire de NimesNimesLanguedoc-RoussillonFrance; 18 Service de GastroentérologieCHU Saint EloiMontpellierFrance; 19 Department of Gastroenterology and Digestive Oncology 26935 Hospital CochinParisFrance; 20 Department of Gastroenterology and Endoscopy 55647 Hôpital Européen Georges PompidouParisÎle-de-FranceFrance; 21 Department of Gastroenterology and Hepatology 26990 CHU BrestBrestBretagneFrance; 22 Department of Gastroenterology and HepatologyNouvel Hopital CivilStrasbourgFrance; 23 Department of Gastroenterology and EndoscopySaint Antoine University HospitalParisFrance; 24 Department of Gastroenterology and EndoscopyHopital PontchaillouRennesBretagneFrance; 25 Department of Gastroenterology and EndoscopyNouvel Hopital CivilStrasbourgFrance; 26 Departmento Gastroenterologie 55647 Hopital Europeen Georges PompidouParisFrance; 27 Department of Gastroenterology and EndoscopyDupuytren University HospitalLimogesFrance; 28 Department of Gastroenterology and Endoscopy 36609 Hôpital Edouard HerriotLyonAuvergne-Rhône-AlpesFrance

**Keywords:** endoscopy lower GI tract, endoscopic resection (polypectomy, ESD, EMRc, …), quality and logistical aspects, preparation, polyps/adenomas/…

## Abstract

**Introduction** The European Society of Gastrointestinal Endoscopy has highlighted the environmental impact of endoscopic practice and the need to reduce it. One of the major contributors is patient transportation, despite the availability of remote alternatives for preprocedural consultations. The aim of this study was to compare face-to-face (FF) and virtual remote (VR) consultations prior to colorectal endoscopic submucosal dissection (ESD) in terms of effectiveness, safety, and carbon footprint.

**Methods** We used a prospective national database including consecutive colorectal ESD cases performed in France between July 2023 and September 2024. Preprocedural consultations were conducted either FF or via VR consultation, according to patient preference and logistical feasibility. Procedure-related travel included diagnostic colonoscopy and/or local consultation, anesthesiology consultation (local or remote, excluding same-day combined visits), gastroenterology consultation at the referral center, and travel for the ESD procedure itself. Environmental impact was estimated based on the distance between the patient’s residence and the endoscopy unit, the mode of transportation, and validated conversion coefficients.

**Results** Between July 2023 and September 2024, 1729 patients undergoing colorectal ESD were included; 1451 (84%) underwent face-to-face (FF) and 278 (16%) virtual remote (VR) preprocedural consultations. Baseline characteristics were comparable. En bloc, R0, and curative resection rates were similar between FF and VR groups (97% vs 95%, *p* = 0.31; 92% vs 88%, *p* = 0.13; 90% vs 90%, *p* = 0.86), as were bleeding and perforation rates. The median cumulative travel distance was significantly lower in the VR group (180.0 km [IQR 261.0] vs 394.6 km [IQR 447.6], *p* < 0.001). Median cumulative carbon footprint decreased from 89.4 to 31.9 CO₂-eq units ( *p* < 0.001), corresponding to a total saving of 51,106 CO₂-eq units.

**Conclusion** Colorectal ESD performed after VR consultation shows comparable effectiveness and safety to FF consultation, while significantly reducing the carbon footprint.

## Introduction

Endoscopic submucosal dissection (ESD) is an established curative treatment for superficial gastrointestinal neoplasia, enabling en bloc resection of large lesions regardless of size. 1 However, ESD is technically demanding, time-consuming, and associated with procedure-related risks. Endoscopy represents the third-largest source of hospital waste, 2 and the European Society of Gastrointestinal Endoscopy (ESGE) has issued a position statement highlighting the environmental impact of endoscopic practice and the need to reduce its carbon footprint. 3 Among the major contributors to carbon emissions, patient transportation plays a particularly relevant role. 4 Replacing in-person outpatient visits with virtual consultations may substantially reduce travel-related emissions. In this context, careful patient selection and a thorough preprocedural consultation are essential to optimize clinical outcomes while minimizing environmental impact. Traditionally, such consultations are performed face-to-face, but telemedicine has emerged as a valuable alternative, particularly following the COVID-19 pandemic. 5 6 7 Telemedicine reduces barriers to care for geographically distant patients, 8 9 lowers healthcare costs, and decreases environmental impact. 10 11 12 In gastroenterology, it is used for remote patient education, tele-expertise in diagnostic imaging, home-based capsule endoscopy pathways, telemonitoring of inflammatory bowel disease, and virtual multidisciplinary tumor boards in oncology. 11 12 13 14 Despite its widespread adoption, evidence regarding teleconsultation specifically before advanced endoscopic procedures such as ESD remains limited.

This multicenter prospective study aimed to compare outcomes of patients undergoing colorectal ESD after either teleconsultation (virtual remote consultation [VR]) or face-to-face (FF) consultation, focusing on procedural effectiveness, safety, and environmental impact.

## Methods

### Study Population and Outcomes

This prospective multicenter study included consecutive patients who underwent colorectal ESD between July 2023 and September 2024 across 18 different centers. Patients were assigned to either VR or FF consultation based on personal preference and logistical feasibility. VR consultations were performed using secure, GDPR-compliant telemedicine platforms. Given the multicenter design, the technical infrastructure was not fully standardized and ranged from basic video consultation systems to more integrated hospital-based telemedicine platforms allowing real-time access to electronic medical records. In all centers, teleconsultations ensured synchronous audio–video interaction between the patient and the physician. When available, endoscopy reports, images, and histological results were reviewed in real time via screen sharing or were transmitted in advance through secure channels. Consultations were conducted by the endoscopist, with optional nurse assistance when needed, particularly for elderly or frail patients. The content of the VR consultation was standardized across centers and included: (i) review of clinical history and comorbidities; (ii) discussion of lesion characteristics based on prior endoscopy reports and imaging; (iii) assessment of indication for ESD; (iv) explanation of the procedure, including risks and benefits; and (v) preprocedural instructions, including anticoagulant management and bowel preparation. Patients requiring physical examination or presenting with uncertain indications were preferentially scheduled for FF consultation ( **Fig. S1** ). The overall VR group included any patient who underwent at least one preprocedural consultation remotely, either with the gastroenterologist, the anesthesiologist, or both. Demographic and clinical variables collected included sex, age at inclusion, antiplatelet or anticoagulant therapy, and ASA physical status. Endoscopic variables included lesion location, lesion size (mm), and CONNECT classification. 15 Procedural effectiveness was assessed according to en bloc resection, R0 resection, and curative resection rates. Complications were defined according to international consensus criteria 16 and included both intraprocedural and postprocedural adverse events. Bleeding was defined as delayed post-ESD bleeding requiring blood transfusion, rehospitalization or treatment, whereas perforation included both intraprocedural and delayed perforations identified during follow-up. Procedure duration (minutes) and dissection speed (mm ^2^ /min) were also recorded. The primary aim of the study was to compare the effectiveness of VR and FF consultations by evaluating compliance with ESGE quality indicators for colorectal ESD (en bloc resection and curative resection rates). 1 Secondary aims were to compare safety outcomes, including adverse events, and procedural performance metrics (procedure duration and dissection speed), as well as to assess the environmental impact of each consultation pathway by quantifying CO₂-equivalent (CO₂e) emissions avoided through reduced patient travel.

### Environmental Impact Calculation

Environmental impact was calculated by estimating the CO₂e emissions associated exclusively with patient transportation along the care pathway. Procedure-related trips considered in the analysis included: (i) initial transport for diagnostic colonoscopy and/or local consultation; (ii) travel for preprocedural consultations related to the ESD (anesthesiology and gastroenterology), when not performed on the same day; and (iii) round-trip travel for the ESD procedure at the distant expert center.

For each patient, the distance between the home address and the endoscopy center (round trip) was obtained using standardized mapping tools. The mode of transportation (car, ambulance, light medical vehicle, airplane, or public transport) was recorded. CO₂ emissions of the travels were computed by multiplying the distance travelled by the corresponding emission factor (kg CO₂e per kilometer) for each transportation mode. Emission factors were derived from ADEME Carbon Base, 17 a validated and widely used French national database for environmental impact calculation. This database provides standardized conversion coefficients based on real-world fuel consumption and life-cycle assessment models, ensuring robust and reproducible estimates.

For patients in the VR group, the environmental benefit was calculated as the CO₂ emissions avoided by preventing an additional in-person round trip to the hospital for the consultation. For the FF group, total emissions corresponded to the actual travel required for the preprocedural visit. All results were expressed in kilograms of CO₂e and aggregated to estimate the overall environmental savings attributable to teleconsultation.

### Statistical Analysis

Statistical analyses were performed using standard inferential methods. Categorical variables were compared using the chi-square test or Fisher’s exact test when expected cell counts were <5. Continuous variables were assessed for normality using the Shapiro–Wilk test and presented as mean ± standard deviation (SD) or median with interquartile range (IQR), as appropriate. Group comparisons were performed using Student’s *t* test for normally distributed variables and the Mann–Whitney *U* test for non-normally distributed variables.

Missing data were handled using complete-case analysis. All statistical tests were two-sided, and a *P* -value < 0.05 was considered statistically significant.

## Results

Between July 2023 and September 2024, 1729 patients were included in the study; among these, 1451 patients were informed by the endoscopists through FF consultations (84%) and 278 (16%) through VR consultations.

**Fig. 1:**
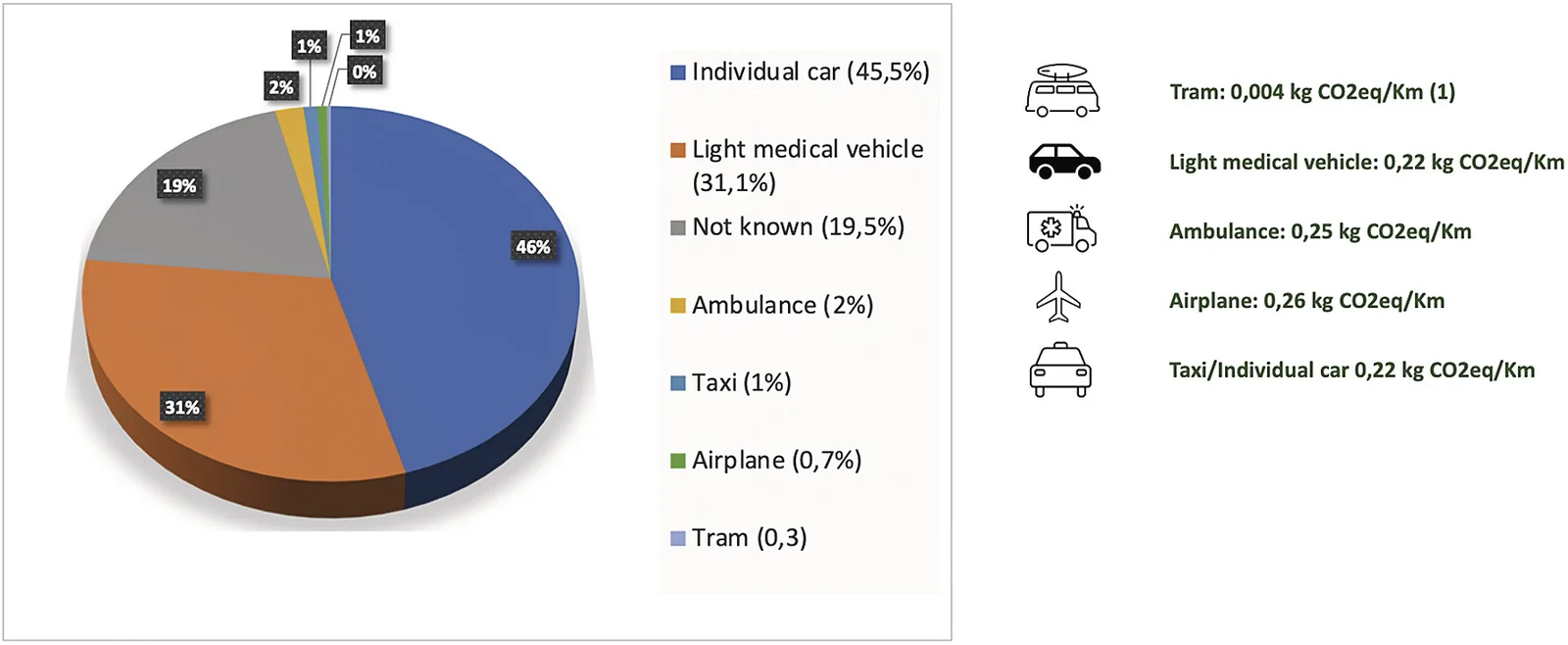
Transportation mode for the consultations.

The anesthesiology consultation was performed in the reference distant center for 1331, (77%) patients and in the local center for 349 patients (10%); in 8 cases (0.5%), it was not performed. In 4 patients, this data was not available. In 801 out of 1,331 (60%) patients undergoing FF consultation in the reference center, the anesthesiology consultation took place on the same day as the endoscopy consultation. In all, 35 (2%) patients underwent both endoscopy and anesthesiology consultations in VR mode. No patient underwent a virtual anesthesiology consultation without a concomitant virtual gastroenterology consultation. The type of transportation used to reach the consultation is shown in Fig. 1 : 45.5% ( *N* = 787) traveled by private car, 31.1% ( *N* = 538) by light medical vehicle, 2% ( *N* = 86) by ambulance, 1% ( *N* = 17) by taxi, 0.7% ( *N* = 12) by airplane, and only 0.3% ( *N* = 5) by tram. For the remaining patients, the mode of transportation was not known.

The two groups (FF and VR) were comparable in terms of clinical characteristics (age, use of anticoagulant therapies, ASA score, and body mass index) and lesion characteristics (morphology and size) ( Table 1 ).

**Table 1:** Baseline clinical and lesion characteristics according to preprocedural consultation modality (virtual remote [VR] vs face-to-face [FF]).

Characteristics	VR group ( *N* = 278)	FF group (=1451)	*P* Value
Age			
*Median (IQR – range)*	70 (13; 34–92)	71 (11; 19–95)	0.27
ASA score, *n* (%)			
I–II	117	709	0.07
III–IV	55	237	
	*N* = 172	*N* = 946	
Body Mass Index, kg/m ^2^			
*Median (IQR – range)*	26.1 (8.3; 16.2–50.7)	25.9 (6.3; 14.5–64.8)	0.20
Use of anticoagulants, *n* (%)			
Yes	17	137	0.19
No	166	911	
	*N* = 193	*N* = 1048	
Lesion morphology, *n* (%)			
Polypoid lesion	46	294	0.41
Nonpolypoid lesion	110	622	
Subepithelial lesions	2	27	
	*N* = 158	*N* = 943	
Lesion size			
*Median (IQR – range)*	40 (20; 10–130)	40 (20; 10–200)	0.06

### ESD Outcomes

**Fig. 2:**
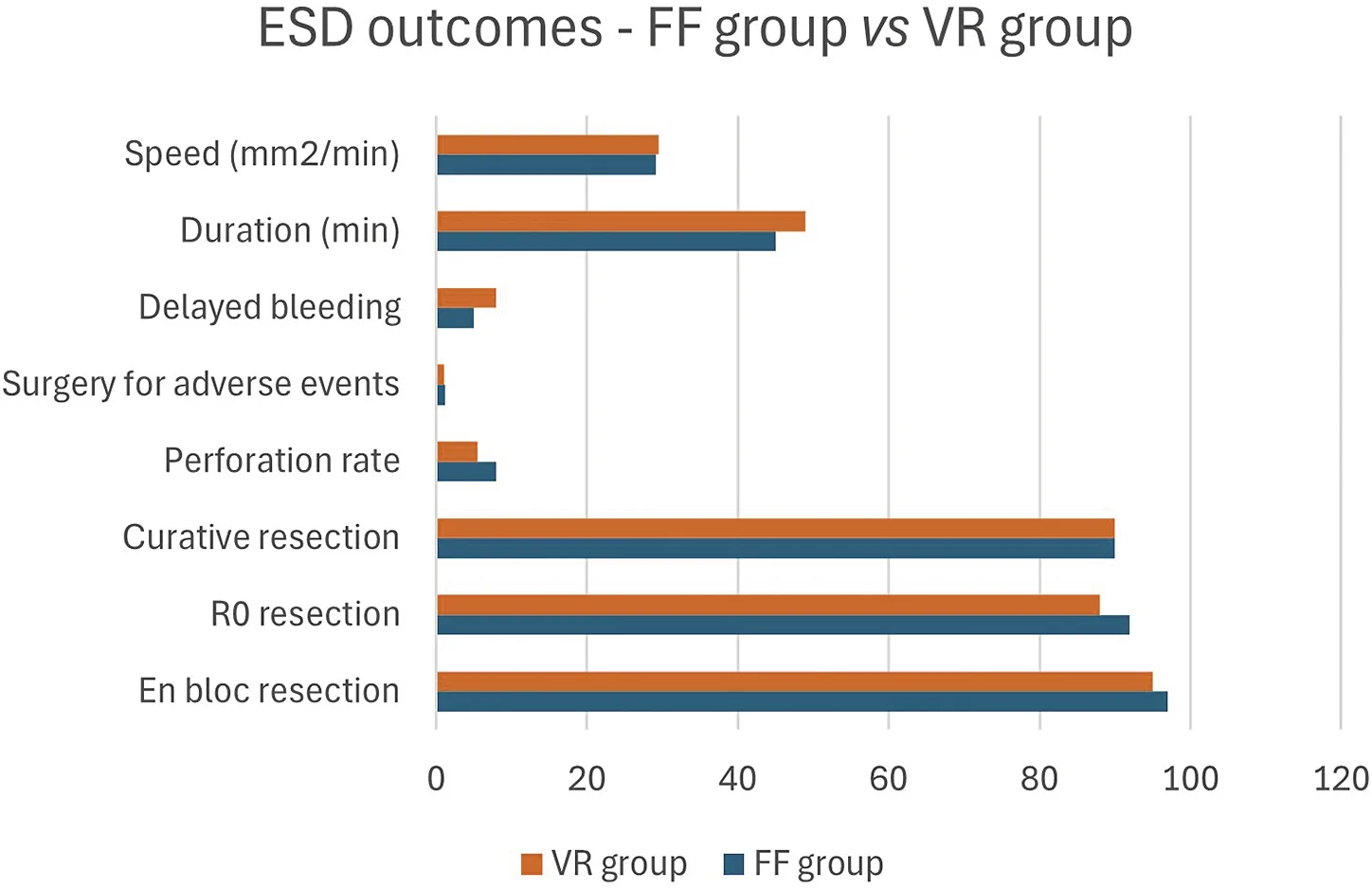
Comparison of endoscopic submucosal dissection (ESD) outcomes between the virtual consultation (VR) group and the face-to-face (FF) group. Bars represent procedure speed (mm ^2^ /min), procedure duration (min), en bloc resection rate, R0 resection rate, curative resection rate, perforation rate, need for surgery due to adverse events, and delayed bleeding rate.

En bloc resection was achieved in 97% of cases in the FF group and 95% in the VR group ( *p* = 0.31). R0 resection rates were 92% in the FF group and 88% in the VR group ( *p* = 0.13). Curative resection rates were identical between the two groups, with 90% in both the FF and VR groups ( *p* = 0.86). Regarding safety outcomes, the bleeding rate was 5% in the FF group and 8% in the VR group ( *p* = 0.11), while perforation occurred in 8% of FF cases and 5.5% of VR cases ( *p* = 0.22). The median duration of procedure was 45 minutes (IQR 45) in FF group and 49.5 minutes (IQR 51.3) in VR group ( *p* = 0.08). The median speed was 29.4 mm ^2^ /min (IQR 20.7) in the FF group and 29.1 mm ^2^ /min (IQR 24.6) in VR group ( *p* = 0.21). Overall, ESD outcomes in terms of both effectiveness and safety were comparable across the two consultation modalities, as illustrated in Fig. 2 . From an anesthesiologic perspective, no procedure-related mortality was observed in either the FF or VR group. ICU admission occurred in 1 patient in the VR group and 11 patients in the FF group; however, this variable was affected by a substantial amount of missing data (143 patients in the VR group and 676 patients in the FF group), precluding reliable comparative analysis. The median length of hospital stay was 1 day in both groups (VR: IQR 1–2, range 1–4; FF: IQR 1–2, range 1–15).

### Patient Travels

Data on patient travel distances were available for 1336 of the 1729 included patients (77%). Patients attended the expert center a median of 1.5 times (range 1–5). Considering all travel related to the care pathway, including the diagnostic colonoscopy at the local center, the anesthesiology consultation (either locally or at the expert center, when not performed on the same day of the gastroenterology visit), the gastroenterology consultation (local or expert center), and the ESD procedure at the expert center, the median cumulative distance travelled in the overall population was 382.9 km (IQR 194.9–643.0 km). A total of 1258 (94%) patients underwent an in-person (FF) preprocedure gastroenterology consultation, whereas 77 (6%) had a VR consultation. The median cumulative distance was 394.6 km (IQR 447.6 km) in the FF group and 180.0 km (IQR 261.0 km) in the VR group ( *p* < 0.001). Globally, the median distance between the patient’s residence and the local center was 37.10 ± 83.08 km ( *n* = 1619), while the distance from residence to the expert center was 117.20 ± 500.95 km.

### Carbon Footprint

Carbon footprint data were available for 1294 of the 1729 (75%) included patients: 1061 (82%) who underwent an in-person (FF) preprocedure gastroenterology consultation and 233 (18%) who underwent a VR consultation. The median cumulative carbon footprint of the transports was 89.4 CO₂-e (IQR 48.0–145.1) in the FF group and 31.94 CO₂-eq (IQR 6.73–66.04) in the VR group ( *p* < 0.001).

The VR gastroenterology consultation resulted in a median CO₂-e saving of 64.24 units (IQR 26.84–104.72), corresponding to a total reduction of 51,106 CO₂-eq units across the study population. In addition, virtual anesthesiology consultations accounted for an additional 25,295 CO₂-e units saved.

An exploratory missingness analysis was performed comparing patients with and without available CO₂e data. No differences were observed in the main clinical outcomes ( **Table S1** ).

## Discussion

This prospective multicenter study demonstrates that teleconsultation prior to ESD is safe and effective, without compromising procedural outcomes. Key ESGE quality indicators were met in both groups, and no significant differences were observed in adverse event rates, suggesting that virtual consultation does not compromise procedural outcomes in this highly specialized setting. Importantly, the adoption of teleconsultation was associated with a substantial reduction in environmental impact, corresponding to a cumulative saving of 51,106 CO₂-eq units across the study population, mainly driven by the avoidance of patient travel.

These results are in line with growing evidence across gastroenterology and hepatology showing that telemedicine can maintain quality of care while improving efficiency and reducing indirect costs. Teleconsultation has proven effective in capsule endoscopy, 14 18 inflammatory bowel disease monitoring and follow-up, 19 20 multidisciplinary management of digestive oncology, 13 21 and hepatology, where it preserves adherence to hepatocellular carcinoma surveillance programs and facilitates timely transplant listing. 22 Similar benefits have been reported in pediatric gastroenterology, where VR consultations significantly reduce time without impairing care quality. 23

Together, these data support the broader applicability of telemedicine in complex gastroenterological pathways. Nevertheless, concerns regarding patient comprehension, communication, and shared decision-making during teleconsultations remain relevant for technically demanding procedures such as ESD. In this context, previous studies from other medical specialties have demonstrated a high concordance between virtual and in-person consultations. 9 10 11 12 13 24 Moreover, structured teleconsultation pathways, including nurse-assisted visits or standardized educational materials, may help overcome digital literacy barriers and ensure adequate patient understanding, especially in the elderly or frail populations.

From an environmental perspective, health care systems represent a major and growing source of CO₂ emissions with patient transportation accounting for a substantial proportion of this burden. 3 Regarding the high proportion of the travel impact in the total footprint of a procedure or strategy, describing precisely the use of telemedicine is a key point of future research in the field.

Minimizing avoidable travel for highly specialized consultations therefore constitutes a concrete and immediately actionable strategy to lower the environmental footprint of advanced endoscopy. In this regard, teleconsultation aligns well with emerging sustainability initiatives within endoscopy units, offering measurable environmental benefits without sacrificing safety or procedural effectiveness.

Despite its prospective multicenter design and the specific focus on environmental outcomes, this study has several limitations. The groups were unbalanced, and allocation to VR or FF consultation was based on patient preference and logistical feasibility rather than randomization, introducing the possibility of selection bias and residual confounding. The environmental analysis focused exclusively on patient transportation, which is considered one of the major contributors to healthcare-related emissions, but it did not account for the total environmental footprint of the ESD pathway. Digital literacy was not specifically assessed. However, no significant differences in age were observed between the virtual and FF consultation groups despite the imbalance in sample size; nevertheless, other factors such as educational level or access to technology were not evaluated and may still represent a source of selection bias. In addition, patient satisfaction, comprehension, and perceived quality of communication were not formally assessed. Operator expertise, center experience, and local procedural strategies were not systematically recorded and therefore could not be adjusted for. As these factors are known determinants of ESD outcomes, residual confounding remains possible despite the comparable baseline characteristics and procedural outcomes observed between groups. Finally, although en bloc and curative resection rates were included as effectiveness outcomes, these indicators are primarily influenced by procedural and operator-related factors rather than by the consultation modality itself. Nevertheless, these endpoints were included to ensure that the use of teleconsultation was not associated with poorer clinical outcomes resulting from inappropriate patient selection or treatment planning.

In conclusion, in this multicenter observational study, VR consultation before colorectal ESD was associated with reduced travel-related carbon emissions, without significant differences in observed procedural outcomes compared with FF consultation. Given the nonrandomized design and potential residual confounding, these findings should be interpreted cautiously and warrant confirmation in future larger studies with more homogeneous populations.

## Supplementary Material

Ergänzendes Material Supplementary Material
