# Supplementary material for: Impact of Teleconsultation before Endoscopic Submucosal Dissection on Quality of Care and Environmental Sustainability: A French Multicenter Study
Source: Endosc Int Open. 2026 Aug 19;14:a29320269. doi: 10.1055/a-2932-0269 (PMC13494681; doi:10.1055/a-2932-0269)
Supplement: Supplementary file 1 [file 10-1055-a-2932-0269_29336174.pdf]

Impact of teleconsultation before endoscopic submucosal dissection on quality of care and environmental sustainability: a French multicenter study

Elena De Cristofaro1 ; Amina Abdulle 2; Vincent Lépilliez3; Bertrand Brieau4; Thibaut Degand5; Jérôme Rivory6; Alexandru Lupu6, Florian Rostain6; Frédéric Moll6; Jean-Baptiste Chevaux7; Romain Legros8; Nicolas Musquer9; Hugo Lepetit8; Sarah Leblanc3; Marion Schaefer7; Yann Le Baleur10; Raphaelle Grau 6; Geoffroy Vanbiervliet 11; Edouard Chabrun12; Arthur Berger13; Philippe Leclercq14; Ludovic Caillo15; Antoine Debourdeau16; Paul Doumbe-Mandengue17; Gabriel Rahmi18; Lucille Quénéhervé 19; Lucille Héroin20; Xavier Dray21; Timothée Wallenhorst22; Joël Lacroute20 ; Ariane Vienne18 ; Jérémie Jacques8; Mathieu Pioche6.

Table S1: Missingness analysis for CO_2e data availability

Variable	CO_2e data available (n=1,294)	CO_2e data unavailable (n=435)	p value
Age, median (IQR), years	69.6 (62.4–75.4)	70.9 (62.9–76.2)	0.194
ASA III–IV, n/N (%)	220/852 (25.8)	72/266 (27.1)	0.746
BMI, median (IQR), kg/m²	26.1 (23.2–29.4)	25.4 (22.8–29.1)	0.198
Antiplatelet therapy, n/N (%)	138/897 (15.4)	50/334 (15.0)	0.928
Anticoagulant therapy, n/N (%)	115/897 (12.8)	39/334 (11.7)	0.658
Lesion size, median (IQR), mm	30.0 (25.0–40.0)	30.0 (25.0–40.0)	0.687
Non-polypoid lesion, n/N (%)	562/820 (68.5)	170/281 (60.5)	0.017
En bloc resection, n/N (%)	867/898 (96.5)	320/333 (96.1)	0.836
R0 resection, n/N (%)	617/687 (89.8)	245/260 (94.2)	0.05
Delayed bleeding, n/N (%)	44/688 (6.4)	8/250 (3.2)	0.084
Perforation, n/N (%)	70/856 (8.2)	21/312 (6.7)	0.488
Surgery for complication, n/N (%)	7/683 (1.0)	0/251 (0.0)	0.199

Endosc Int Open | DOI 10.1055/a-2932-0269 | © The Author(s).

Fig. S1: Illustration of the pre-procedural pathway for both virtual and face-to-face consultations.

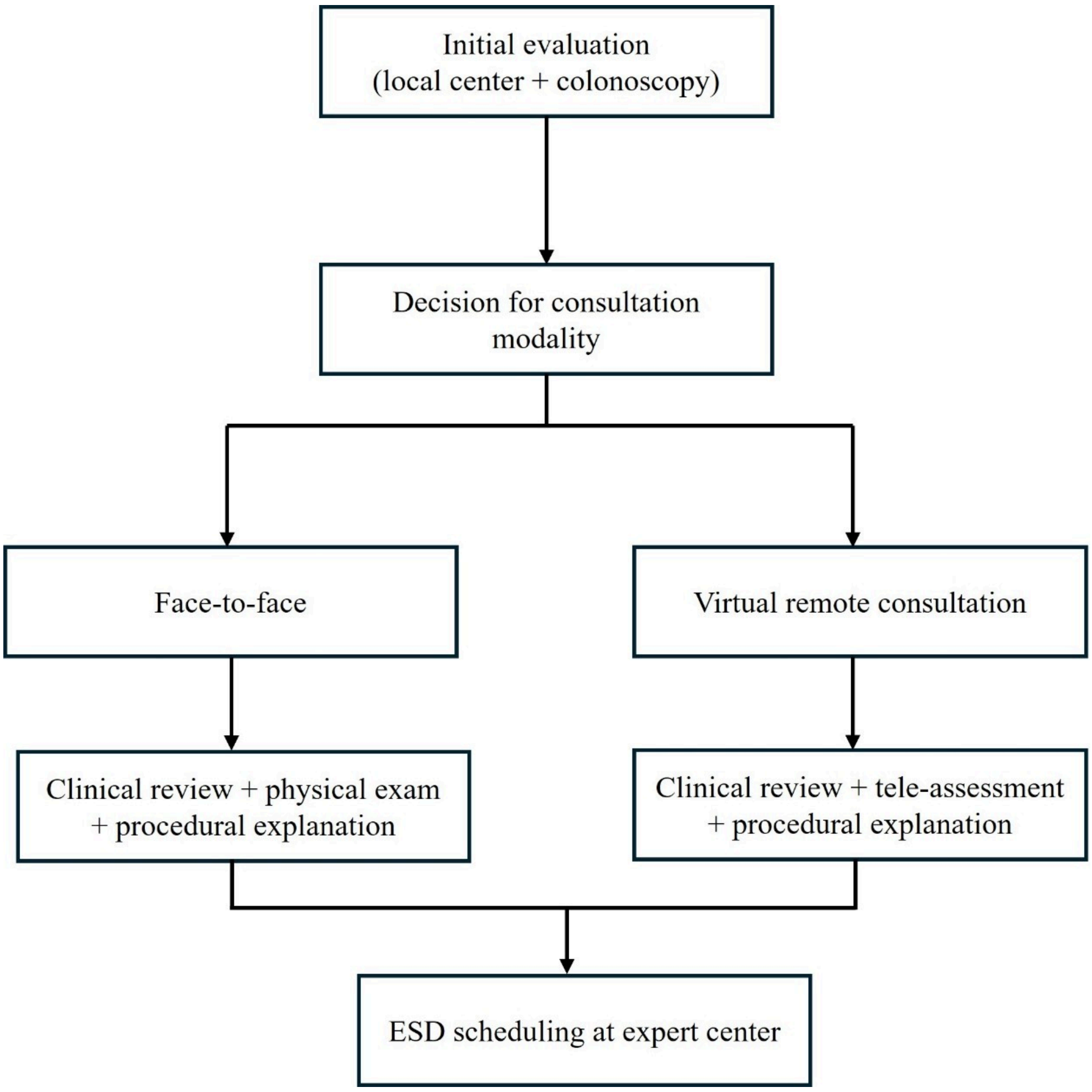

Endosc Int Open | DOI 10.1055/a-2932-0269 | © The Author(s).
